# Clinical and Radiographic Gingival Thickness Assessment at Mandibular Incisors: an Ex Vivo Study

**DOI:** 10.3290/j.ohpd.a44925

**Published:** 2020-07-24

**Authors:** Andreas Gkogkos, Dimitrios Kloukos, George Koukos, George Liapis, Anton Sculean, Christos Katsaros

**Affiliations:** a Periodontist, Department of Periodontology, 251 Hellenic Air Force & VA General Hospital, Athens, Greece. Hypothesis, performed the clinical measurements, wrote the manuscript.; b Senior Lecturer, Department of Orthodontics and Dentofacial Orthopedics, School of Dental Medicine, University of Bern, Switzerland; Orthodontis, Department of Orthodontics and Dentofacial Orthopedics, 251 Hellenic Air Force & VA General Hospital, Athens, Greece. Idea, experimental design, proofread the manuscript.; c Periodontist, Department of Periodontology, 251 Hellenic Air Force & VA General Hospital, Athens, Greece. Performed the clinical measurements and statistical analysis.; d Dentist in Private Practice, Athens, Greece. Scanned the mandibles, proofread the manuscript.; e Professor, Department of Periodontology, School of Dental Medicine, University of Bern, Switzerland. Idea, proofread the manuscript.; f Professor, Department of Orthodontics and Dentofacial Orthopedics, School of Dental Medicine, University of Bern, Switzerland. Idea, proofread the manuscript.

**Keywords:** cone-beam computed tomography, gingival biotype, periodontal tissue, ultrasound

## Abstract

**Purpose:**

Gingival phenotype influences the outcomes of various dental procedures. The objective of the current study was to assess the agreement between various clinical and radiographic methods for evaluating gingival thickness.

**Materials and Methods:**

This ex-vivo study evaluated gingival thickness on 20 porcine cadavers. Gingival thickness was assessed at both central mandibular incisors with: a) trans-gingival probing with a standard periodontal probe (PB); b) trans-gingival probing with a stainless steel acupuncture needle (AN); c) ultrasound device (USD); and d) Cone Beam Computed Tomography (CBCT). Intra-examiner reproducibility and method error were also evaluated.

**Results:**

Trans-gingival measurements with the standard PB and the AN were found to be almost identical in gingival thickness assessment (mean GT 1.11 mm vs 1.14 mm for the left incisor and mean GT 1.12 mm vs 1.11 mm for the right incisor, respectively). USD and CBCT yielded values that were statistically significantly higher than AN. Both USD and CBCT values were higher than PB, but this difference was statistically significant only for the left central incisor. Finally, USD values exceeded CBCT measurements, but this difference was not statistically significant. There was no evidence of systematic differences between the repeated CBCT measurements (p = 0.06 for the left incisor and p = 0.55 for the right incisor).

**Conclusions:**

CBCT measurements proved to be highly repeatable and comparable to the USD measurements, while there were some indications that both CBCT and USD measurements were systematically higher than either PB or AN.

Gingival phenotype is considered a significant parameter for all dental specialists, as it plays a key role in the success of periodontal plastic surgery,^9,17,18^ implant placement,^22,24,27^ restorative dentistry or prosthodontics^25^ and orthodontics.^7,38^ Gingival thickness (GT) appears to be one of the most important factors when determining gingival phenotype;^38^ GT has been reported to present a stronger association with gingival phenotype, rather than gingival width or the height of the papilla.^11,16^

Several methods and definitions have been proposed to classify gingival phenotype, but most of them have been found to be unclear and sometimes inconsistent.^39^ Although visual assessment of gingival phenotype is a simple and frequently used technique, it cannot be considered a reliable method to identify gingival phenotype due to the subjectivity of the examiner.^14^

For the objective quantitative measurement of GT, a variety of methods have been implemented, trans-gingival probing with a periodontal probe being one of the most commonly used methods. Although it is a simple, straightforward technique, it is considered invasive, as it is routinely performed under local anaesthesia. Furthermore, the local anaesthetic used may increase gingival volume, thus affecting the outcome measure.^33^ To overcome this problem, an ultrasound device with high reproducibility in GT measurements was introduced as an alternative, non-invasive method.^13,28^ In a recent clinical study in orthodontic patients, trans-gingival probing with the periodontal probe or ultrasound determination seemed to be reliable choices for everyday practice.^23^

The periodontal probe has also been used for optical GT evaluation, based on the transparency of the periodontal probe through the soft tissue after probe placement in the gingival sulcus.^11,21^ Finally, the evolution of Cone-Beam Computed Tomography (CBCT) devices has led to low radiation emission, and in several studies has been used as a standard method for determining not only bone thickness, but GT as well.^1,8,17,29^ It has been reported that CBCT presents high diagnostic accuracy in assessing GT, with minimal discrepancy between clinical and radiographic measurements.^17^ However, evidence-based data to confirm the precision of this method in assessing soft tissue thickness is still limited.

Thus, the aim of this ex-vivo study was to assess GT with four different methods, including two methods of trans-gingival probing, ultrasound assessment, and CBCT imaging, and to explore the agreement among them and the repeatability of CBCT measurements.

## Materials and Methods

### Animal Model and Study Design

Previous studies have indicated porcine gingiva as a validated animal model to resemble human gingiva.^36,37^ Among the reasons for their relative comparability are the similarities in gross anatomy and physiology in relation to human tissue, as well as the economic and ethical advantages. Porcine head cadavers of 20 6-month-old pigs, slaughtered 14 h prior to the experiments, were obtained from a slaughterhouse. Cadaver heads were transported and stored at 4°C, and attention was given to their state of hydration throughout the experimental period by spraying them with 0.9% saline solution.^26^

### Clinical Parameters

A periodontist (GK) assessed the GT of mandibular incisors. Measurements were carried out at both central mandibular incisors, mid-facially on the buccal aspect of each tooth, and 2 mm apically to the free gingival margin, with the 4 following methods.

#### Trans-gingival probing with a standard periodontal probe (PB)

Measurements were performed by perpendicularly inserting a probe (CPU 15 UNC, Hu-Friedy; Chicago, IL, USA), equipped with a silicone stopper, into the soft tissue until resistance was felt (i.e. hitting the root surface or the buccal bone). Then, the distance between the silicone stopper and the probe tip (i.e. GT) was measured with a digital caliper (OEM, Maxwell Tools; Hangzhou, China) to an accuracy of 0.01 mm.

#### Trans-gingival probing with a sterile, spring-handled, stainless-steel, disposable acupuncture needle (AN)

In transgingival probing with a 0.18-mm-diameter AN (Dongbang, DB102; Seoul, Korea), care was taken to apply a light force, thus restricting needle penetration to the gingiva and avoiding penetrating the alveolar bone. Measurements were performed by perpendicularly inserting the needle mounted with a silicone stopper in the soft tissue, until the alveolar bone was reached. Subsequently, GT was measured with a digital caliper, as above. The rationale of implementing a second trans-gingival method was the difference between the diameter of the penetration instruments and therefore the force one has to apply in order to penetrate the gingiva.

#### Ultrasound

Measuring GT with an ultrasound device (USD) (Krupp SDM, Austenal Medizintechnik; Cologne, Germany) is based on the ultrasonic pulse-echo-principle: ultrasonic pulses are transmitted through the sound-permeable tissue (1518 m/s), and are reflected at the surface of the hard tissue. By timing the received echo, GT is determined and digitally displayed. Measurements may range between 0.5 and 8.0 mm with a resolution of 0.1 mm. The ultrasonic frequency was 5 MHz and the diameter of the transducer probe 3 mm, with a weight of 19 g. The first author (AG) performed all USD measurements.

#### Cone-Beam Computed Tomography (CBCT)

All porcine head cadavers underwent CBCT examination in a private dental clinic in Athens, Greece. CBCT images were acquired using the Carestream CS 8100 3D Imaging System (Carestream Health; Rochester, NY, USA) at 85 kV and 5 mA for 15 s and a single 360-degree image rotation. The CBCT scans were obtained with an 8 x 9 cm field of view and 150 μ voxel size. Images were processed by CS 3D image software (Carestream Health). During the examination, the lip was retracted in order to enable imaging of labial soft tissues. The GT in all CBCT images was measured by one author (DK) in duplicate with an intermediate interval of one month in order to evaluate the intra-examiner repeatability. No adjustment in the contrast of the CBCT images was performed.

### Method Error Assessment (Accuracy)

Digital caliper measurements were tested for possible method error. PB and AN were mounted with a silicone stopper set at a 3-mm length, defined with the Florida Probe System (Florida Probe; Gainesville, FL, USA). Then, the distance of the stopper to the tip of both the PB and AN were repeatedly measured with the caliper 10 times by the same clinician (GK). We calculated the difference between the measurements and the true value (3 mm) for both the PB and the AN, and the average of the differences of the 10 measurements was used to estimate the method error (bias) of each method. Bias was employed as a measure of accuracy. The null hypothesis that bias was zero was tested with paired t-tests between the measurements from each method and the true value. Accuracy was assessed using the standard deviation of the above measurements.

### Statistical Analysis

Descriptive statistics were applied for PB, AN, USD and CBCT gingival thickness measurements.

#### CBCT repeatability assessment (Intra-examiner method error)

The repeatability of each method was initially evaluated graphically using scatter plots of the replicates vs the indices of the samples.^12^ Thorough repeatability assessment was conducted using the methodology of Bland and Altman.^5,6^ Bias was assessed with paired t-tests between repeated measurements, while precision was assessed with the repeatability coefficient. The relationship of the differences between the replicates and the magnitude (average between the replicates) was assessed both graphically (via Bland-Altman plots) and with the Spearman's rank correlation coefficient.

#### Method comparison / method agreement

Each of the following GT measuring techniques AN, USD and CBCT were graphically compared to PB by means of the Bland-Altman analysis. Paired t-tests were conducted to assess the mean difference between PB and each of the methods AN, USD and CBCT, as well as to estimate the respective 95% confidence interval (95% CI). Each method comparison analysis was concluded after calculating the 95% Limits of Agreement (95% LOA) and the corresponding 95% CIs of the LOA.

All normality assumptions were evaluated both graphically and statistically (Shapiro-Wilk test). Statistical significance was set at α = 5%. All statistical analyses and plots were conducted using Stata 13.0/SE software (Stata; College Station, TX, USA).

## Results

In the 20 porcine head cadavers, two missing central mandibular incisors (one left and one right) left 38 central incisors for evaluation. The descriptive statistics for PB, AN, USD and CBCT gingival thickness measurements are reported in Table 1.

**Table 1:** Descriptive statistics of all gingival thickness measurements (mm)

	Mean (SD)	Min	Max
**Mandibular left central incisor**			
PB	1.11 (0.17)	0.83	1.40
AN	1.14 (0.24)	0.78	1.57
USD	1.33 (0.25)	1.00	2.00
CBCT	1.28 (0.26)	0.80	1.90
**Mandibular right central incisor**			
PB	1.12 (0.24)	0.74	1.58
AN	1.11 (0.22)	0.70	1.49
USD	1.24 (0.25)	0.70	1.70
CBCT	1.23 (0.28)	0.80	1.90

### Method Error Assessment (Accuracy)

**Fig 1:**
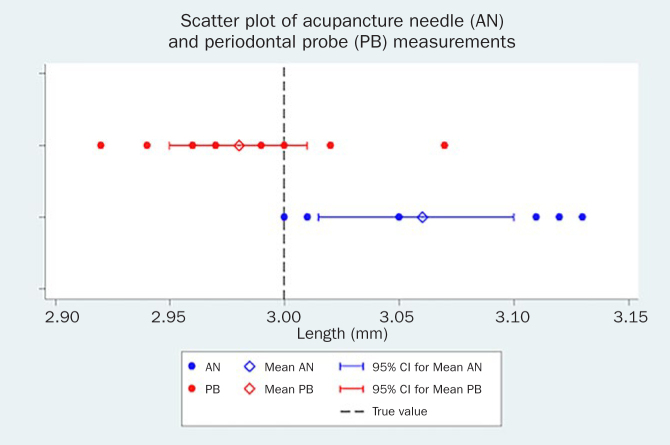
Scatter plot of AN and PB measurements.

Figure 1 represents the scatter plot of the ten PB and the ten AN measurements on the 3-mm standard length. Overall, measurements with the periodontal probe were found to be more accurate than those with the acupuncture needle. There is no evidence that the PB measurements on the standard 3-mm length are biased (bias = -0.02 mm, SD = 0.04 mm, 95% CI = -0.05 mm, 0.01 mm, p = 0.24). On the other hand, AN presented a positive difference from the true value with a mean 0.06-mm greater estimation (SD = 0.06 mm, 95% CI = 0.02 mm, 0.10 mm, p = 0.01). Nevertheless, clinical relevance is arguable.

### CBCT Repeatability Assessment (Intraexaminer Method Error)

The results of the paired t-tests for bias between the 1st and the 2nd CBCT measurements are reported in Table 2.

**Table 2:** Repeatability assessment: results of the paired t-test between the repeated CBCT measurements (bias = 2nd minus 1st measurements [in mm])

	Bias (SE)	95% CI	p-value
**Mandibular left central incisor**	-0.05 (0.02)	(-0.10, 0.01)	0.06
**Mandibular right central incisor**	-0.02 (0.03)	(-0.07, 0.04)	0.55

**Fig 2:**
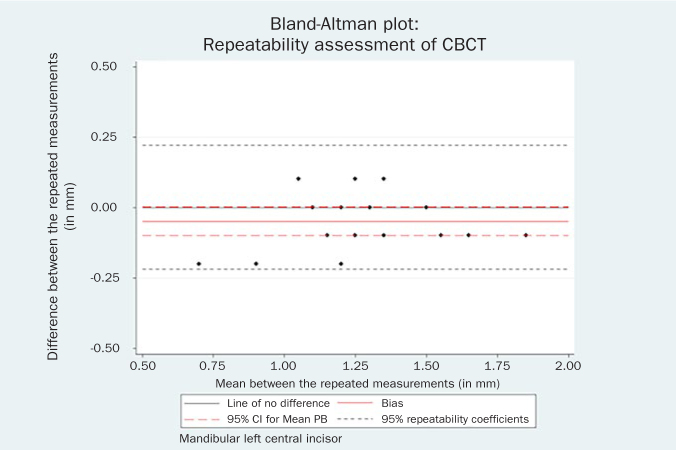
Bland-Altman plot of the repeated CBCT measurements for the mandibular left central incisor.

**Fig 3:**
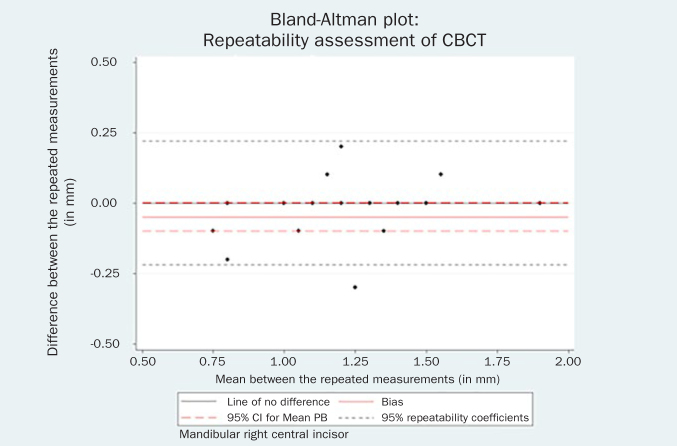
Bland-Altman plot of the repeated CBCT measurements for the mandibular right central incisor.

Statistical analysis indicated that the repeated measurements for both the mandibular left central incisor (bias = -0.05 mm, 95% CI = -0.10, 0.01, p = 0.06) and the mandibular right central incisor could be considered identical (bias = -0.02 mm, 95% CI = -0.07, 0.04, p = 0.55). The respective Bland-Altman plots are displayed in Figs 2 and 3.

### Method Comparison / Method Agreement

The results of the paired t-test between PB and each of the methods AN, USD and CBCT along with the respective 95% CIs are reported in Table 3.

**Table 3:** Method comparisons: results of the paired t-tests (mean difference, SE, 95% CI, p-value) between all methods used for GT assesment (PB, AN, USD and CBCT) with the corresponding 95% LOA and the respective 95% CI for the LOA (in mm)

	Mean difference (SE)	95% CI	p-value
**Mandibular left central incisor**			
AN – PB	0.03 (0.05)	(-0.08, 0.13)	0.58
USD – PB	0.22 (0.06)	(0.10, 0.34)	0.01
CBCT – PB	0.18 (0.05)	(0.07, 0.28)	0.01
USD – AN	0.19 (0.06)	(0.06, 0.32)	0.01
CBCT – AN	0.14 (0.06)	(0.03, 0.26)	0.02
CBCT – USD	-0.04 (0.05)	(-0.15, 0.07)	0.44
**Mandibular right central incisor**			
AN – PB	-0.01 (0.05)	(-0.12, 0.10)	0.84
USD – PB	0.12 (0.07)	(-0.02, 0.26)	0.09
CBCT – PB	0.11 (0.06)	(-0.01, 0.23)	0.08
USD – AN	0.06 (0.05)	(0.00, 0.26)	0.05
CBCT – AN	0.12 (0.04)	(0.03, 0.21)	0.01
CBCT – USD	-0.01 (0.05)	(-0.12, 0.10)	0.84

The respective Bland-Altman plots are displayed in Figs 4–9 and method agreement plots in Figs 10–15.

**Fig 4:**
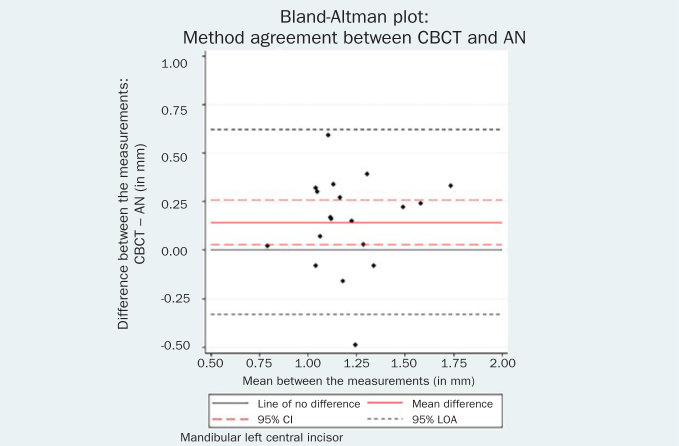
Bland-Altman plot CBCT vs AN 31.

**Fig 5:**
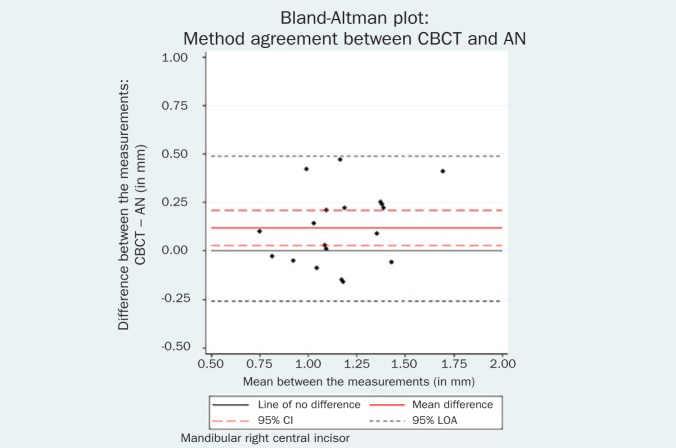
Bland-Altman plot CBCT vs AN 41.

**Fig 6:**
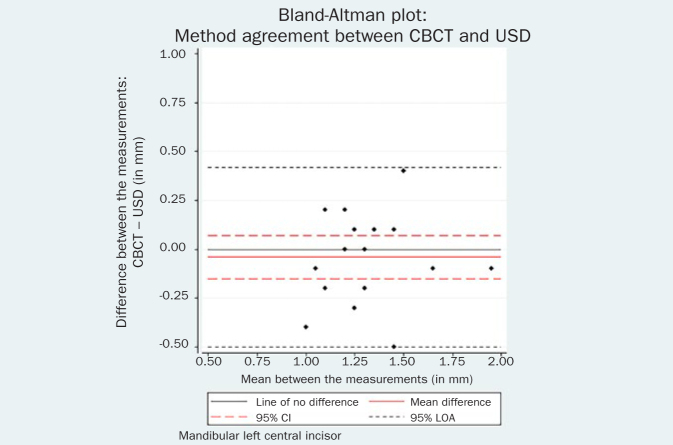
Bland-Altman plot CBCT vs USD 31.

**Fig 7:**
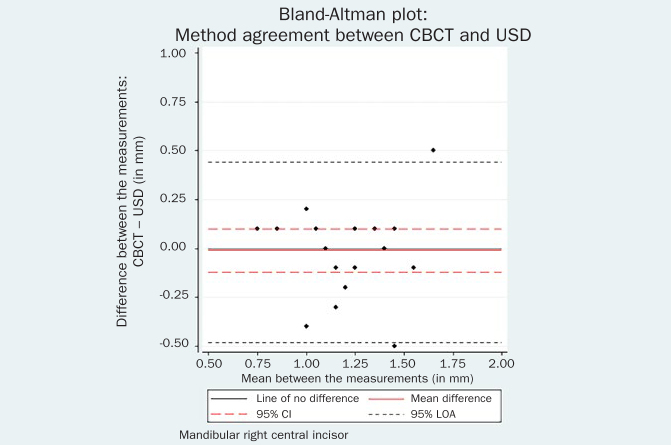
Bland-Altman plot CBCT vs USD 41.

**Fig 8:**
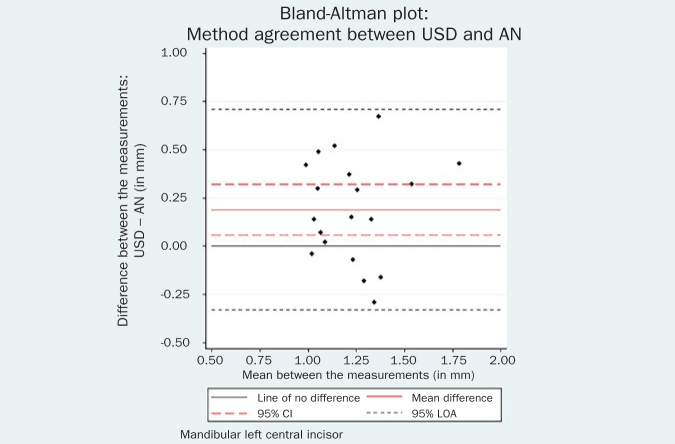
Bland-Altman plot USD vs AN 31.

**Fig 9:**
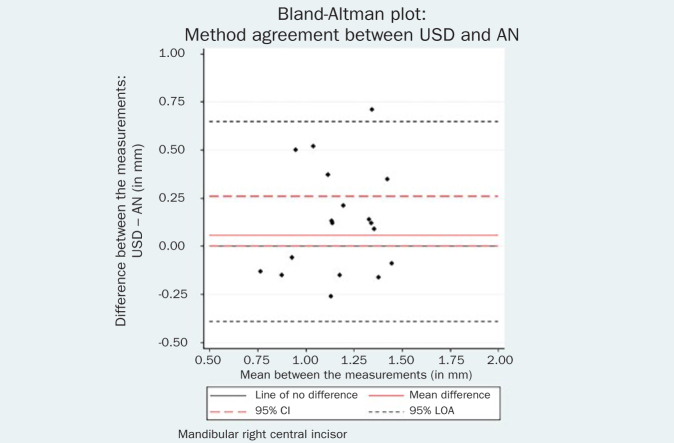
Bland-Altman plot USD vs AN 41.

**Fig 10:**
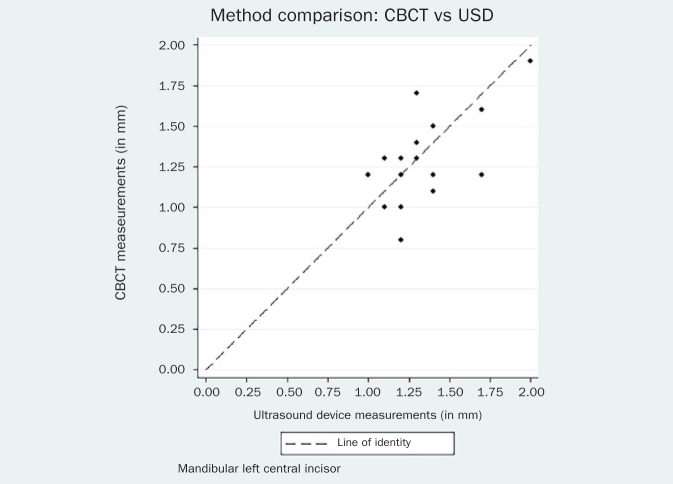
Method agreement plot CBCT vs USD 31.

**Fig 11:**
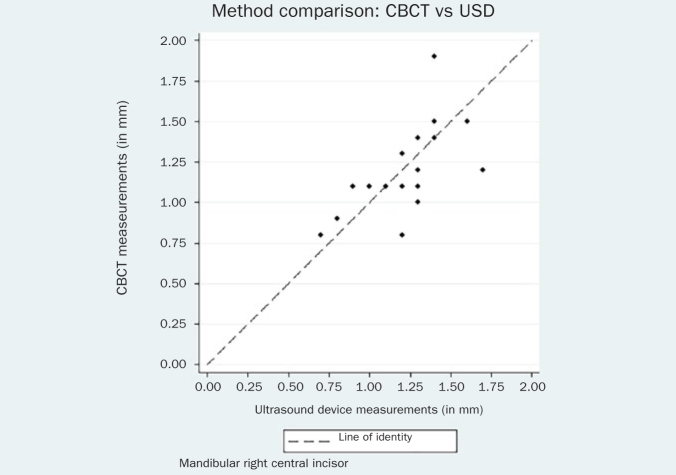
Method agreement plot CBCT vs USD 41.

**Fig 12:**
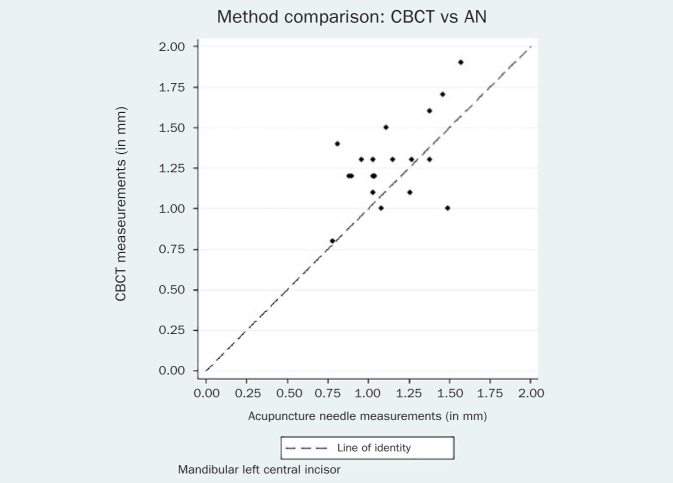
Method agreement plot CBCT vs AN 31.

**Fig 13:**
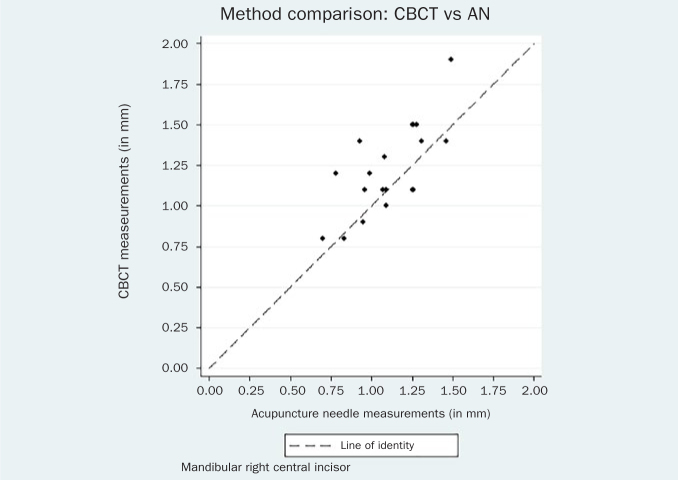
Method agreement plot CBCT vs AN 41.

**Fig 14:**
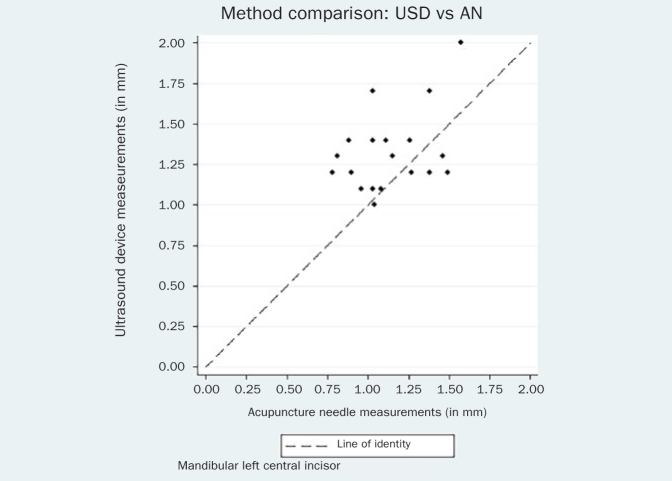
Method agrseement plot USD vs AN 31.

**Fig 15:**
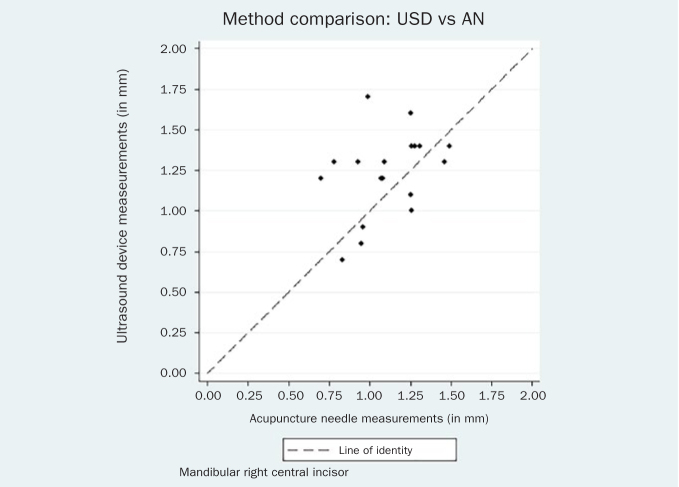
Method agreement plot USD vs AN 41.

## Discussion

Since gingival phenotype appears to influence the outcomes of various dental procedures, including periodontal, implant, and orthodontic treatment, its precise measurement is important for treatment planning. Assessment of gingival phenotype should involve an easy and reproducible method for distiguishing between ‘thin’ and ‘thick’ gingivae.

It must be pointed out that even slight differences in GT may prevent accurate identification of high-risk patients in regard to their soft tissue thickness. Specifically, patients with thick gingiva (GT > 0.8 mm or 1 mm) are relatively resistant to gingival recession after surgical and/or restorative treatment.^2,3,15,31^ On the contrary, patients with a thin-scalloped phenotype have been associated with compromised soft-tissue healing following surgical and/or restorative treatment.^2,3,15,24,30-32^ These findings clearly indicate the need for a preventive method for these high-risk patients before various interventions involving the gingiva.

The purpose of this study was to assess GT with four different methods used in clinical practice, by focusing on mandibular anterior teeth, since it is an area of great concern regarding aesthetics and function in relation to recession development, especially after change of tooth inclination or position. We decided to use porcine central mandibular incisors only, in order to determine any possible association of these teeth with human incisors that exhibit localised and multiple recessions after orthodontic treatment. Moreover, practical reasons for using these teeth included their flat anatomy, which facilitated the application of the USD transducer.

Visual inspection is a highly subjective method for determining gingival phenotype.^10,14^ Therefore, direct measurement is considered the most objective method for GT assessment, although its clinical application is associated with some challenges, since it involves penetration of the gingival tissue with sharp instruments.^23^ In the present study, two invasive methods were selected: trans-gingival probing with either a PB or an AN.

In addition, a direct, non-invasive method was employed: USD, mainly owing to its high reproducibility.^13,20,21,28^ Finally, CBCT imaging was selected, as it is an indirect, non-invasive method for GT measurement and it has been reported to have a high diagnostic accuracy.^4,17^

Regarding method agreement, the expected difference between AN, PB, USD and CBCT measurements was not zero. The comparison between PB and AN methods showed no evidence of systematic difference for either the left (mean difference 0.03 mm, 95% CI = -0.08, 0.13, p = 0.58) or the right (mean difference -0.01 mm, 95% CI = -0.12, 0.10, p = 0.84) mandibular incisors. These results imply that both penetration methods presented comparable precision of GT measurement.

USD values were statistical significantly higher than AN values for both left (mean difference 0.19 mm, 95% CI 0.06, 0.32, p = 0.01) and right (mean difference 0.06 mm, 95% CI 0.00, 0.26, p = 0.05) central incisor. The same tendency towards larger USD measurements was noted between USD and PB. Additionally, CBCT measurements were systematically higher than the AN for both left (mean difference 0.14 mm, 95% CI 0.03, 0.62, p = 0.02) and right (mean difference 0.12 mm, 95% CI 0.03, 0.21, p = 0.01) central incisor.

The comparison between USD and PB methods showed no evidence of systematic difference for the right mandibular incisor (mean difference 0.12 mm, 95% CI -0.02, 0.26, p = 0.09). However, the USD measurements were significantly higher than the PB measurements by 0.22 mm (95% CI = 0.10, 0.34, p = 0.001) for the contralateral tooth. Although there is a statistical difference, the actual difference in GT measurement between these two methods may be regarded as clinically similar. This confirms previous findings in a comparable human study.^23^

The comparison between CBCT and PB methods showed no evidence of systematic difference for the right mandibular incisor (mean difference 0.11 mm, 95% CI -0.01, 0.23, p = 0.08). Again, the CBCT measurements were significantly higher than the PB measurements by 0.18 mm (95% CI = 0.07, 0.28, p = 0.01) for the contralateral tooth.

The comparison of the two non-invasive methods for GT assessment, the difference between USD and CBCT measurements was not zero, but it was not statistically significant. CBCT measurements were slightly lower than USD, but the clinical significance was unimportant (for the left incisor: mean difference -0.04 mm, 95% CI -0.15, 0.07, p = 0.44; for right incisor: mean difference -0.01 mm, 95% CI -0.12, 0.10, p = 0.84).

Finally, according to the present results, there was no evidence of systematic differences between the repeated CBCT measurements for either the left or right mandibular incisors (p = 0.06 and 0.55, respectively).

When interpreting the results of the present study, it should be kept in mind that in contrast to the porcine mandible, where anterior teeth are mostly well aligned, the results may not necessarily be applicable to all tooth areas in human subjects, since accessibility or local anatomic factors (such as tooth crowding or inclination) can influence the clinical handling of the various instruments. Moreover, ultrasound possesses a resolution of 0.1 mm, whereas calipers had an accuracy of 0.01 mm when measuring PB and AN. This may be regarded as a potential limitation in the use of USD.

As far as CBCT images are concerned, it should be borne in mind that they have a certain degree of inaccuracy attributed primarily to image generation, processing, voxel size and various types of artefacts that might be present. In general, the smaller the voxel size, the higher the precision/resolution of the information provided. Larger voxels may include different tissues, and thus, the subsequent grayscale value may not clearly visualise one specific tissue, such as bone. This issue is primarily evident at the limits between neighbouring tissue types of different radiodensity. However, at the same time, the smaller the voxel size, the greater are motion artefacts. Thus, based on the above considerations and also on the need to keep radiation exposure to a minimum, a specific CBCT image can only reach a certain degree of detail in terms of the information it provides.^34,35^

Finally, it should be highlighted that the routine assessment of GT by means of CBCT might not always be justified, due to the associated amount of radiation in humans.^18^ The ‘as low as reasonably achievable’ (ALARA) principle, which is fundamental to the principles of radiation protection, should be applied.

## Conclusions

CBCT measurements proved to be highly repeatable and comparable to the USD measurements, while there were some indications that both CBCT and USD measurements were systematically higher than both PB or AN.
